# Emergencies in cardiovascular surgery

**DOI:** 10.1186/1749-8090-8-S1-O2

**Published:** 2013-09-11

**Authors:** Z Mitrev, T Anguseva

**Affiliations:** 1 Special Hospital for Surgery Fillip II, Skopje, Macedonia

## Background

Urgent surgery is part of a daily practice on cardiovascular units, and it may be required in patients with conditions such as acute coronary syndrome, endocarditis and valvular diseases, trauma, acute aortic dissection as well as acute vascular embolisations and Leriche syndrome.

## Methods

In emergency situation, preoperative patient work-up for cardio-vascular surgery is quite different from the elective setting. Since 03/2000 till 01/2012 we have analyzed a consecutive series of 12 172 cases out of which 2214 underwent emergency procedures (18.19%). The most frequent problems requiring urgent intervention were thoracic aortic aneurysms (448 cases; 20.24%); coronary artery disease (1006 cases; 45.47%) abdominal aortic aneurysms (125 cases - 66 with rupture; 2.96%), peripheral vascular (364 cases; 16.46%), and others (329 cases: 14.87%).

## Results and discussion

Urgent thoracic and abdominal aortic aneurysm repair accounted for 23% respectively and the corresponding proportion for peripheral vascular surgery is 16%. However, urgent surgery for acute coronary ischemia, valvular and congenital heart disease accounted for somewhat less than 60% for each group of these pathologies. Mortality rate was 43 (1.91%).

## Conclusion

Systematic pre-operative diagnostic work-up is a recognized tool for procedure related risk assessment and superior management of diseases. However, hemodynamic instability and other time related events correlated with negative outcome, are the main driving forces for accelerated diagnostic pathways.

